# Limitations of current chemotherapy and future of nanoformulation-based AmB delivery for visceral leishmaniasis—An updated review

**DOI:** 10.3389/fbioe.2022.1016925

**Published:** 2022-12-14

**Authors:** Prakash Kumar, Pawan Kumar, Nidhi Singh, Salil Khajuria, Rahul Patel, Vinod Kumar Rajana, Debabrata Mandal, Ravichandiran Velayutham

**Affiliations:** ^1^ Department of Biotechnology, National Institute of Pharmaceutical Education and Research, Hajipur, India; ^2^ National Institute of Pharmaceutical Education and Research, Ahmedabad, India; ^3^ National Institute of Pharmaceutical Education and Research, Kolkata, India

**Keywords:** amphotericin B, nanoparticle, drug delivery, leishmaniasis, clinical status

## Abstract

Visceral leishmaniasis (VL) is the most lethal of all leishmaniasis diseasesand the second most common parasiticdisease after malaria and,still, categorized as a neglected tropical disease (NTD). According to the latest WHO study, >20 Leishmania species spread 0.7–1.0 million new cases of leishmaniasis each year. VL is caused by the genus, *Leishmania donovani* (LD), which affects between 50,000 and 90,000 people worldwide each year. Lack of new drug development, increasing drug resistance, toxicity and high cost even with the first line of treatmentof Amphotericin B (AmB), demands new formulation for treatment of VLFurther the lack of a vaccine, allowedthe researchers to develop nanofomulation-based AmB for improved delivery. The limitation of AmB is its kidney and liver toxicity which forced the development of costly liposomal AmB (AmBisome) nanoformulation. Success of AmBisome have inspired and attracted a wide range of AmB nanoformulations ranging from polymeric, solid lipid, liposomal/micellar, metallic, macrophage receptor-targetednanoparticles (NP) and even with sophisticated carbon/quantum dot-based AmBnano delivery systems. Notably, NP-based AmB delivery has shown increased efficacy due to increased uptake, on-target delivery and synergistic impact of NP and AmB. In this review, we have discussed the different forms of leishmaniasis disease and their current treatment options with limitations. The discovery, mechanism of action of AmB, clinical status of AmB and improvement with AmBisome over fungizone (AmB-deoxycholate)for VL treatment was further discussed. At last, the development of various AmB nanoformulation was discussed along with its adavantages over traditional chemotherapy-based delivery.

## Graphical Abstract

**Figure:**
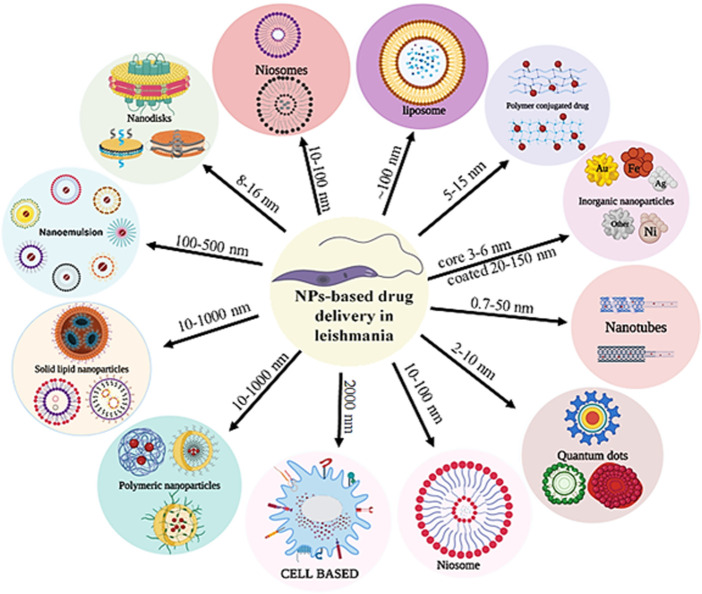
The nanoparticles with different size, shape and structure for drug delivery against *Leishmania donovani*.

## Introduction

Leishmaniasis is caused by a protozoan parasite of the Leishmania genus (family Trypanosomatidae) transmitted by the bites of infected phlebotomine sandflies (Steverding, 2017). This disease has been reported in 89 countries across Africa, Asia, the Americas, and the Mediterranean. This parasite has infected 12–15 million people globally, with 350 million at risk. About 1.5-2 million new cases are diagnosed annually, with 70,000 deaths (Torres-Guerrero et al., 2017). Changes in the natural environment or changes in the human host due to geographical diversity make them susceptible to infection by the vectors as malnutrition associated immunosuppression isone of the major factors for this disease (Desjeux, 2001).

## Types of leishmaniasis

There are several diseases caused by the Leishmania parasite in humans, including visceral leishmaniasis (VL), cutaneous leishmaniasis (CL), mucocutaneous leishmaniasis (MCL), and post kala-azar dermal leishmaniasis (PKDL) (Choi and Lerner, 2001; Von Stebut, 2015).

### Visceral leishmaniasis

The LD complex causes VL (van Griensven and Diro, 2012; Singh et al., 2016) and LD is a protozoan parasite that causes leishmaniasis in Asia and Eastern Africa (Alves et al., 2018; Selvapandiyan et al., 2019). It mainly infects young and immunocompromised people (Chappuis et al., 2007). Untreated VL is frequently fatal (Dantas-Torres and Brandão-Filho, 2006). Every year, the WHO reports between 50,000 and 90,000 new cases of VL, where it is estimated that only 25%–45% people report to the clinic (Dantas-Torres and Brandão-Filho, 2006; Chappuis et al., 2007; van Griensven and Diro, 2012). However, 90% of cases were in India, Nepal, Bangladesh, Sudan, Brazil, and Ethiopia (Dantas-Torres and Brandão-Filho, 2006; Singh et al., 2016; Alves et al., 2018). The WHO classified VL as a NTD in 2015 because of low public awareness (Bi et al., 2018) and constantlack of funding/research for its treatment although it is ranked second in death and fourth in morbidity (Singh et al., 2016). VL is classified as Zoonotic or Anthroponotic based on the vector which transmits the disease (Chappuis et al., 2007; Alves et al., 2018). Anthroponotic VL is transmitted from human to human, whereas Zoonotic VL is passed from animal to animal (Chappuis et al., 2007). VL has an incubation period of 10 days to 1 year (Saporito et al., 2013). Fever, weight loss, hepatosplenomegaly, and pancytopenia or anemia is symptoms of VL (Saporito et al., 2013; Alves et al., 2018). Infection of the reticuloendothelial system causes hepatomegaly (Saporito et al., 2013). In India, almost 98% cases of VL are in the eastern state of Bihar.

### Cutaneous leishmaniasis

CL is a neglected tropical disease whose global prevalence increased by 174.2 percent from 1990 to 2013 (Aronson and Joya, 2019). It is a major health issue in 88 countries where it is prevalent (Meireles et al., 2017). 90 percent of instances occurred in Afghanistan, Algeria, Brazil, Iran, Peru, Saudi Arabia, and Syria (de Vries et al., 2015). *Leishmania major* causes CL in the old world, whereas*Leishmania tropica* causes CL in the new world (Reithinger et al., 2007). Commonly affected organs are the face, forearms, and lower legs (Bilgic-Temel et al., 2019). CL can manifest as ulcerative, chronic, nodular, hyperkeratotic, psoriasis form, plaque-like, or verrucous (Magill, 2005). An initial skin lesion may be overlooked or misdiagnosed which allows spreading of this disease (Reithinger et al., 2007). DiffusedCL is rare and frequently coupled with mucous membrane involvement (Meireles et al., 2017). Acute CL starts as a tiny papule, then ulcerates, enlarges, and forms a volcano-shaped moist lesion (Reithinger et al., 2007). Infection usually occurs in the summer and the illness is continued till the winter (Bilgic-Temel et al., 2019).

### Mucocutaneous leishmaniasis

MCL develops when a cutaneous infection appears to have been cured or under treatment (Handler et al., 2015). MCL is caused by *Leishmania panamensis, Leishmania guyanensis, and Leishmania amazonesis* (Handler et al., 2015). It is mostly linked to *Leishmania brazileinsis* and *Leishmania amazonesis* in the tropical region of Brazil in South America (Vicente and Falqueto, 2018). In the new world *Leishmania vianniacan* causes MCL (Ronet et al., 2011). On the other hand, it impacts the poor population of 88 countries including Africa, Asia, Europe, and North America (Mistro et al., 2017). The lesion occurs 2 years after cutaneous infection, although it can take 30 years to create the develop the symtom (Handler et al., 2015). MCL is not a life-threatening disease that requires treatment (David and Craft, 2009; Handler et al., 2015). It affects the nose and mouth but can spread to the oropharynx and trachea (Handler et al., 2015). The host immune system and parasite virulence determine MCL progression (David and Craft, 2009) where1-10% of infected patients develop mucosa (David and Craft, 2009).

### Post kala-azar dermal leishmaniasis

PKDL occurs in people with a history of VL and was initially described by U.N. Brahmachari in 1922 (Ganguly et al., 2010; Singh et al., 2015). PKDL is found throughout the Indian subcontinent and Sudan (Ganguly et al., 2010; Mukhopadhyay et al., 2014). Post treatment, 5–60% of patients with VL develop PKDL with a skin lesion (Zijlstra et al., 2003). However, current research shows that it can emerge post VL treatment within 1 year in 36% of patients, and within 0–13 months in 64% cases in Sudan (Zijlstra et al., 2017). In South Asia, adults are more affected than children, whereas, in Sudan, children are more affected than adults (Mukhopadhyay et al., 2014). Clinical features associated with PKDL is a measles-like skin lesion (hypopigmented macules, papules and nodules) first appearing on the face and gradually increasing in size (Ganguly et al., 2010; Mukhopadhyay et al., 2014; Singh et al., 2015). Due to bad looking skin lesions and its sociological problem the patients with PKDL hardly reports to the clinic and, therefore, a complete available dataset of PKDL cases across different continents is much more than the actual cases.

### Early treatment, current therapeutics and their limitation

Antimony’s use as early medicine for treatment of leishmaniasis is extensively established. Early on, Paracelsus promoted antimony (Sb^V+^) as a comprehensive panacea. Plimmer and Thompson discovered the antitrypanosoma activity of sodium and potassium tartrate in the 19th century, kicking off the era of antimony use in the last decades of 20th century. Vianna reported treating CL with trivalent antimonial in 1913, Rogers in India in 1915, and Di Cristina and Caronia in Sicily, subsequently, confirmed efficacy against VL, although the medicine was exceedingly unstable and poisonous due to climatic conditions. Clinical resistance and relapses led India’s shorts to conclude that antimony tartrate was unacceptable. Cole described tartar as an unpleasant drug, with side effects including cough, chest discomfort, and profound depression (Haldar et al., 2011a).

The antileishmanial activity of pentavalent antimonials (Palumbo, 2009) thought to be dependent on the prodrug concept, i.e. conversion of Sb^V+^ form to Sb^III+^ form which is more toxic, leading to a hypothesis that only macrophage residing amastigotes are susceptible to Sb^V+^. The Sb^V+^ is thought to regulate the parasite’s bioenergetics such as beta-oxidation of fatty acids, glycolysis, and ADP phosphorylation. Some papers found non-specific SH-group protein blocking in amastigotes inhibiting DNA topoisomerase-I. Antimony has recently been shown to modify thiol-redox potential, rendering parasites more sensitive to oxidative stress (Palumbo, 2009). The pentavalent antimonials can be given intramuscularly or intravenously and are concentrated in the plasma, liver, and spleen. Its half-life is 2 h. The pentavalent antimonials are biotransformed into trivalent antimonials in the liver and eliminated in the urine over 24–76 h. Total volume of distribution (V_d_) is 0.22 L/Kg of body weight (Palumbo, 2009).

Pentavalent antimonials and their derivatives cause fatal side effects of cardiotoxicity and hepatotoxicity (Kato et al., 2014). Patients’ complaints of arthralgia, myalgia, diarrhoea, abdominal discomfort, dizziness, headache, nausea, fatigue, and other symptoms significantly decreased after stopping antimonial treatment. The responses were assessed using the CTC (Common Toxicity Criteria) (Soto et al., 2004). Resistance to pentavalent antimonials is complex and multifactorial. Due to host’s variable immune response and lack of research defining the target of Sb^V+^ against the parasite, the researchers have had difficulty defining the resistance mechanism although *in vitro*studies have indicated redox homeostasis and thiol synthesis pathways as targets for Sb^V+^ (Jeddi et al., 2011). It has been the standard treatment for VL for almost 50 years. Antimonial resistance has been a problem in India since the 1980s. Further, pentavalent antimonial is known to cause considerable toxicity, including a greater rate of death for VL treatment cases. For the reasons stated above, the need for new medications increased, and several other drugs (AmB, miltefosine, Paromomycin *etc.*) were produced. AmB and its lipid formulations were tried against human trial of VL with AmBisome in 1990 (Palumbo, 2009; Haldar et al., 2011b).

Pentamidineisothionate is effective in Latin America, regardless of disease duration, age, or the number of lesions (Das et al., 2009). Pentamidine is less effective and has a higher recurrence rate than AmB (Das et al., 2009). Intralesional pentamidine injections are preferred over intralesional antimony injections in cases of CL caused by Bolivian *L. braziliensis*, and also against antimony-resistant parasites although the cost is much higher (Soto et al., 2018). Costa claims that pentamidine can cause diabetic mellitus in doses as little as 1 g. Pentamidine therapy for Leishmaniasis requires, therefore, close patient monitoring (Patel et al., 2009; Soto et al., 2018). Even though pentamidine injections are normally highly effective, patients often report mild symptoms such injection site discomfort, nausea, dizziness, abscess formation, abdominal pain, anorexia and glycosuria, malaise, myalgia, hypotension, and headache (Patel et al., 2009). Pentamidine resistance has been observed, however, the mechanism is unknown (Croft et al., 2006). The response rate for pentamidine as a second-line medication for conventional pentavalent antimonial resistant patients in India went from 95% to 70% in a decade (Das et al., 2009). Thus, combination therapy and various modes of administration are widely desired globally to battle resistance, reduce treatment time and unwanted side effects for these drugs (Meheus et al., 2010; Rahman et al., 2017; Alves et al., 2018).

Paromomycin (PMN), commonly known as Aminosidine, is an aminoglycoside-aminoicyclitol antibiotic used to treat VL and CL (Croft et al., 2006). In the 1960s, it was revealed that it could kill Leishmania in a dose of 11 mg/kg by intramuscular (IM) treatment for 21 days against VL. Paromomycin works by targeting protein synthesis machinery of the parasite (Muñoz et al., 2019). Paromomycin side effects include pruritus, erythema, discomfort, oedema, ototoxicity, elevated creatinine and transaminases (Sosa et al., 2013; Sundar et al., 2014a; Sosa et al., 2019). Previous research has reported clinical failures such as illness persistence, exacerbation, or relapse in some patients (Sosa et al., 2013; Soto et al., 2019). Among 120 patients enrolled in studies, 34 developed at least one adverse effect, including anaemia, ear pruritus, hearing impairment, abdominal discomfort, diarrhoea, nausea, dry mouth, peptic ulcer, asthenia, injection site pain, injection site swelling, pyrexia, abscess, ear infection, nasopharyngitis, malnutrition, neck pain, and pyrexia. In an amastigote macrophage assay, isolates from relapse patients were found to have three-to five-fold less susceptible to the PMN medication after therapy than isolates collected before treatment (Croft et al., 2006). To improve efficacy and reduce toxicity, combination treatments or alternate drug delivery mechanisms are required to treat CL and VL, where combination of PMN with AmB or miltefosine were explored (Rahman et al., 2017; Alves et al., 2018; Moradzadeh et al., 2019).

Miltefosine (MTF) and alkylphosphocholine medicines were discovered in the early 1980s (Balasegaram et al., 2012). Miltefosine was discovered to have antileishmanial activity while being explored as an anticancer medication. MTF is the first oral antileishmanial medication to have promising success in endemic areas of India. MTF at 2.5 mg/kg body weight is administered orally for 28 days in most cases. MTF has a half-life of 150–200 h, which enhances the chances of resistance inside the parasite (Tamiru et al., 2016). MTF affects membrane permeability, phospholipid metabolism, signal transduction, fluidity, and lipid composition, but not nucleic acid content (Shirzadi and medicine, 2019). MTF-treated leishmania promastigotes have shown apoptotic-like features although the exact mechanism behind apoptosis-like death is unclear since eukaryotic-like complete apoptotic machinery is absent in LD. MTF has fewer side effects than pentavalent antimonials and AmB and harmful effects are reversible with the withdrawal of medication. The most common side effects were nausea (87%) and vomiting (50%). Reversible hepatotoxicity (15%) and nephrotoxicity (16%) were recorded, including increases inAlanine transaminase (ALT), Aspartate transaminase (AST), Urea, and Creatinine (Sundar and Chakravarty, 2010).

Resistance to miltefosine is growing in India and Nepal during the treatment of VL. The resistance’s underlying principle is unclear. Mutations in LD MTF transporters (LdMT, L856P, T420N, and L832F) have been observed for MTF-resistance *in vitro* and *in vivo*. Reduced absorption, a quicker metabolism, higher efflux, and altered lipid composition are also associated with MTf-resistance. However, the majority of MTF-resistance was studied *in vitro* which lacks the information related to host’s response associated with this resistance. (Dorlo et al., 2012).

Since Leishmaniasis is a complex disease caused by a variety of species and associated with complex host-pathogen interactions, various drugs in combination therapy is used since monotherapy does not work for long duration and often associated with resistance (Trinconi et al., 2014). Combination therapy of first-line medications or with drugs having synergistic effects and drugs with immune-modulators is intensively investigated to overcome VL and associated co-infections (HIV-VL, HIV-TB)since monotherapy is unsuccessful (Khayeka–Wandabwa et al., 2013; Rahman et al., 2017; Fragiadaki et al., 2018; Moradzadeh et al., 2019; Rebello et al., 2019). Because high doses of AmB can cause nephrotoxicity and irreversible renal damage, high doses of AmB are not used for treatment of PKDL. Therefore, AmB can be used with miltefosine to minimize dosage and side effects (Ravis et al., 2013). Furthermore, *in vitro* and *in vivo* studies using tamoxifen and AmB showed that both medications had additive and synergistic effects at low doses. So different anti-leishmanial medicines have favoredcombination therapy than monotherapy (Trinconi et al., 2014).

In some studies, LD promastigotes developed resistance to combinations therapy, particularly in case of MTF + PMN and MTF + sodium stibogluconate. These kinds of resistance of parasites include improved ability to counteract drug-induced ROS and decreased membrane flexibility. Multidrug-resistant parasites gain overall fitness over wild-type parasites under various stress situations, such as nutrient deprivation, heat-shock, pH stress, and hence can survive as intracellular amastigotes longer inside the macrophages (Rebello et al., 2019). Despite the benefits of combination medication, resistance is possible and may be hazardous in VL associated co-infected patients (Kaur and Rajput, 2014). Relapse, side effects, unsatisfactory final cure rates, treatment duration, and drug resistance in drugs treatingVL are still major concerns in current therapeutics, necessitating the use of a strategy other than conventional chemotherapy/combination therapy to overcome the aforementioned complications (Patel et al., 2009; Alves et al., 2018; Rebello et al., 2019). All the FDA approved and under clinical trial drugs are mentioned in the Table 1.

**TABLE 1:** FDA approved and under clinical trial of different formulation of AmB for VL are listed.

S.N.	Formulation	FDA approved (year)	NIH/Clinical trial status	References
1	Amphotericin B	1959	**-**	Hamill, (2013)
2	Amphotericin B deoxycholate	**-**	2008	Lestner et al. (2010)
3	Amphotericin B Lipid Complex (Abelcet)	1995	**-**	Würthwein et al. (2005)
4	Amphotericin B Colloidal Dispersion (Amphotec)	1996	**-**	Hamill, (2013)
5	AmBisome	1997	**-**	Herrada et al. (2021)
6	Amphotericin B fat Emulsion (Amphomul)	**-**	2008 (phase-2)	Faustino and Pinheiro, (2020)
7	Ambisome^®^, Miltefosine, Paromomycin	**-**	2016 (phase-3)	Goswami et al. (2020)
8	liposomal amphotericin B + paromomycin	**-**	2016 (phase-3)	Berman (2015)
9	Single Dose Liposomal Amphotericin B (AMBISOME)	**-**	2017 (phase-4)	Sundar et al. (2015)
10	CombinationAmbisome and Miltefosine	**-**	2019 (phase 3)	Goswami et al. (2020)

General mechanism of AmB binds and affects the fungal/parasitic cell membrane, causing pores to form. It induces ion leakage, metabolic stress, and ultimately fungal/parasitic cell death by disrupting the cell membrane integrity. Due to its stronger affinity for binding with ergosterol than cholesterol, AmBiosome/Fungizone can be selectively delivered to fungal/parasitic cells. Also, transmission occurs best at body temperature (Soto et al., 2004; Kato et al., 2014). Numerous clinical and preclinical studies have shown that amphotericin B is more nephrotoxic. The nephrotoxicity is probably due to AmB interacting with the renal rubles. However, AmBisome is less nephrotoxic than fungozone (AmB deoxycholate), which induces high fevers and chills when infused to patients with longer durations. An increase in proinflammatory cytokinins from CD14 cells and TLR2-medaited signaling could explain these side effects. The AmBisome has been linked to abnormal liver function tests. The mechanism of hepatotoxicity and abnormal liver function tests is unknown (Balasegaram et al., 2012; Tamiru et al., 2016). In another aspects clinically, AmB is given intravenously for 15–20 days at 0.75–1 mg/kg (Stone et al., 2016). Infusion responses such as fever, chills, and occasional toxicities such as myocarditis, nephrotoxicity, hypokalemia demand frequent monitoring and hospitalisation of the patient, eventually increasing the expense of therapy (Shirzadi and medicine, 2019).

### Early days of amphotericin B application and it is mechanism of action

The macrolide AmB was initially used to treat life-threatening systemic fungal infections. AmB’s amphiphilic properties (lipophilic and hydrophilic chains) limits its solubility in water and other organic solvents (Carolus et al., 2020). In a semi-rigid molecule, two groups enriched in polyene and polyol functional groups rotate around the glycosidic bond containing the mycosamine ring. The development of inter and intra molecule hydrogen bonds, as well as AmB-AmB and AmB-sterol interactions, is dependent on the 3D conformation of these moieties (Faustino and Pinheiro, 2020). When AmB interacts with the membrane, it leads to the generation of pores that allow cellular ions/small molecules to release irreversibly. Also, phospholipid/sterol interaction disruption reduces sterol liquefication and promotes membrane fragility. The AmB selectively binds to ergosterol, which is enriched than cholesterol in fungal and leishmania species membranes (Matsumori et al., 2005). The AmB can bind to ergosterol in the parasite membrane, disrupt the membrane showing its antileishmanial properties. AmB also sequesters cholesterol in the host membrane, inhibiting macrophage-parasite interactions.

AmB may potentially operate as an immuno-adjuvant that leads to the production of interferon (IFN), which helps in the activation of antigen-presenting cells such as macrophages (Kamiński, 2014). The resistance mechanism of LD to AmB revealed many differences from a AmB-sensitive strain, including increased membrane fluidity due to cholesta-5,7,24-trien-3-ol (an intermediate in ergosterol biosynthesis pathway) enrichment instead of ergosterol, decreased AmB absorption, and increased drug efflux due to multidrug resistance protein 1 (MDR1) overexpression and elevated levels of reduced thiol (Kristanc et al., 2019). After the discovery of AmB in the 1950s, the FDA approved the first AmB-deoxycholate complex micellar formulation (Fungizone^®^) in the 1960s. The formulation’s high toxicity was attributed to AmB self-association (mainly as a dimer) along with side effects including nephrotoxicity, hypokalaemia, and myocarditis. Consequences of Fungizone treatment require severe side effects resulting constant observation and prolonged hospital stays, increasing therapy costs. As a result, several efforts have been made to create formulations that can safely contain and transport AmB monomers (Cavassin et al., 2021).

AmB binds to ergosterol in fungal and parasitic cell membranes with ∼3 fold more efficacy than cholesterol. After interacting with ergosterol, it causes proton and monovalent cation loss, depolarization, and ATP concentration-dependent cell death (Ghannoum and Rice, 1999; Purkait et al., 2012). AmB also damages cells by generating free radicals, increasing membrane permeability due to production of ROS. Also, AmB stimulates phagocytic cells, which helps engulf and eliminate fungal cells. By disrupting phospholipids in the leishmania membrane, an ionic imbalance occurs which leads to parasite death (Shadab et al., 2017). Mycosamine and ergosterol hydroxyls have been shown to interact electrostatically by hydrogen bonding and create pores in the cell membrane of fungus. The leishmania membrane having similar membrane architecture including ergosterol exhibits a similar effect after AmB treatment. Diverse investigations have been done to validate AmB’s mechanism with different formulations (Baginski et al., 2002). Comparing gold nanoparticle-conjugated AmB (GL-AmB) to conventional AmB indicated enhanced macrophage absorption for GL-AmB. The drug absorption of AmB in its nanoformulation form in human monocyte THP one and human macrophage J774 cells was studied and found to be elevated (Baginski et al., 2002; Kumar et al., 2019a).

**FIGURE 1:**
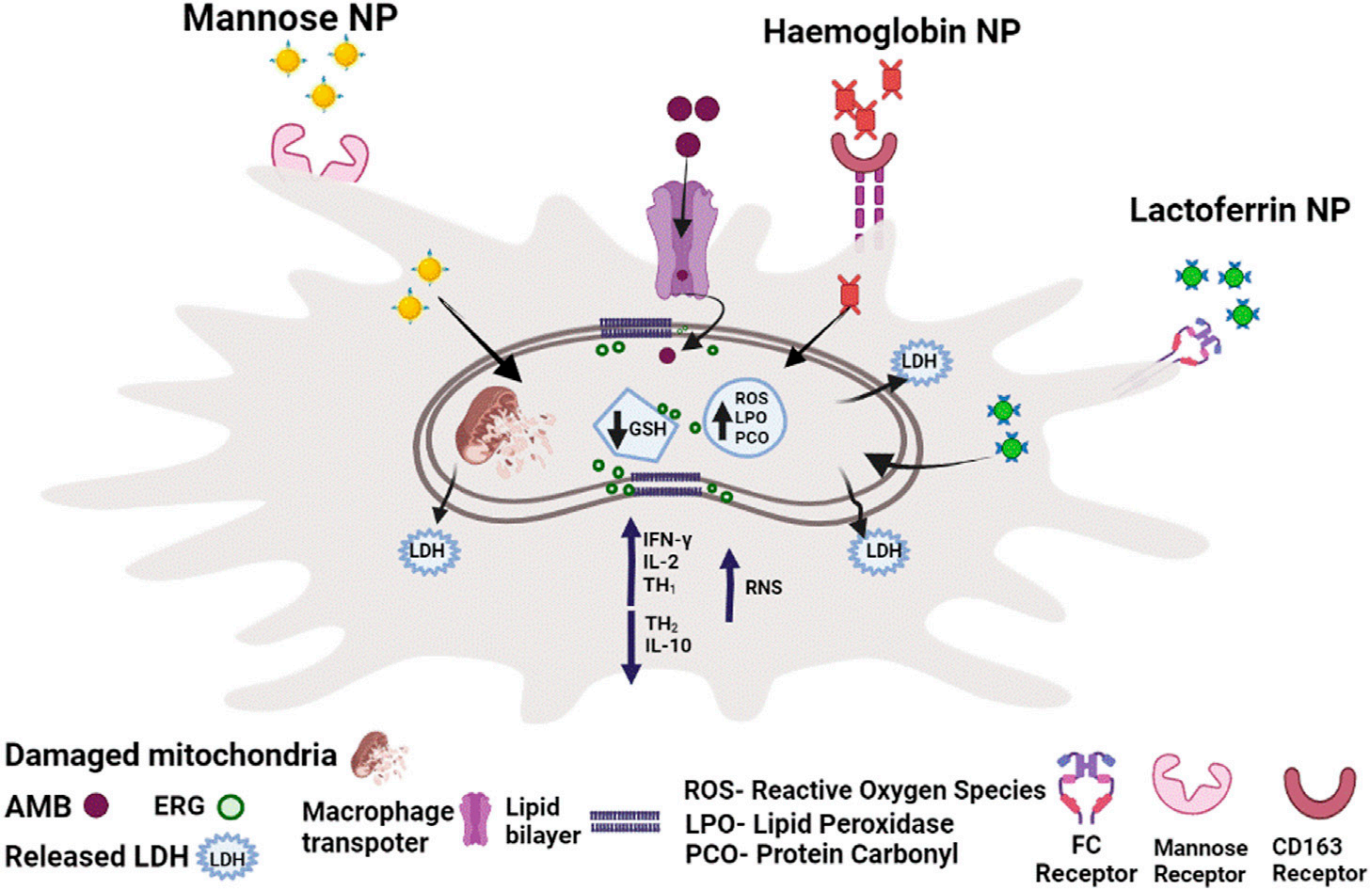
The mechanism of action of AmB against promastigotes and intracellular amastigotes. Depletion of ergosterol, membrane damage with LDH release, disruption of redox homeostasis with reduced thiol content in promastogtes and increased RNS production along with increased Th1/decreased Th2 response in amastigotes reflects AmB-mediated death.

AmB has been linked to an oxidative stress-mediated process as a result of increased drug uptake, membrane damage and lipid peroxidations. ROS is linked to reduced glutathione (GSH), an antioxidant that normalizes cellular redox homeostasis, which was discovered to be substantially lower in cells treated with AmB. The reduced GSH, in turn, reduces the level of trypanathione. Elevated protein carbonylation and lipid peroxidation were also observed, which have been linked to AmB-induced oxidative stress-mediated effect (Figure 1). Trypanathione reductase (TryR) and superoxide dismutase (SOD) relative mRNA levels were significantly lower in AmB-treated cells along with SOD enzyme activity. Inducible nitric oxide synthase (iNOS) expression (involved in macrophage ROS response) was also found up-regulated in AmB-treated amastigotes for GL-AmB than normal AmB (ud Din et al., 2017). The expression level of genes involved in ergosterol pathway was assessed because AmB damages the parasite membrane targeting this specific sterol biosynthesis pathway. The enzyme lanosterol synthase was found to be downregulated in AmB-treated cells. This demonstrates that AmB abolishes parasites by inducing oxidative stress and inhibiting the ERG biosynthesis pathway. Researchers in another study used the lactate dehydrogenase (LDH) release assay to demonstrate membrane damage, which is associated with increased necrosis than apoptosis. VL has previously been linked to a mixed Th1–Th2 cytokine response (Figure 1) where increased Th1 and decreased Th2 response was generally associated with parasite death. Notably, Th1 cytokines (IL-12 and IFN-γ) were upregulated in AmB-treated patients, while Th2 cytokines (IL-10)were downregulated more in GL-AmB treated parasites than normal AmB (Baginski et al., 2002; Kumar et al., 2019a).

### The importance of amphotericin B-induced nephrotoxicity in clinical practice

AmB induced nephrotoxicity can have catastrophic effects in specific patient populations (Safdar et al., 2010). Wingard et al. reported that nephrotoxicity occurred in 53% of patients when treated with AmB for invasive aspergillosis. Patients with AmB-induced renal failure required dialysis in 15% of cases, especially with concurrent use of nephrotoxic medications like cyclosporine. Dialysis was required 38% of the time for those with a creatinine level greater than 2.5 mg/dl. Dialysis was also connected to a threefold increase in the chance of death of those patients (Wingard et al., 1999).

**FIGURE 2:**
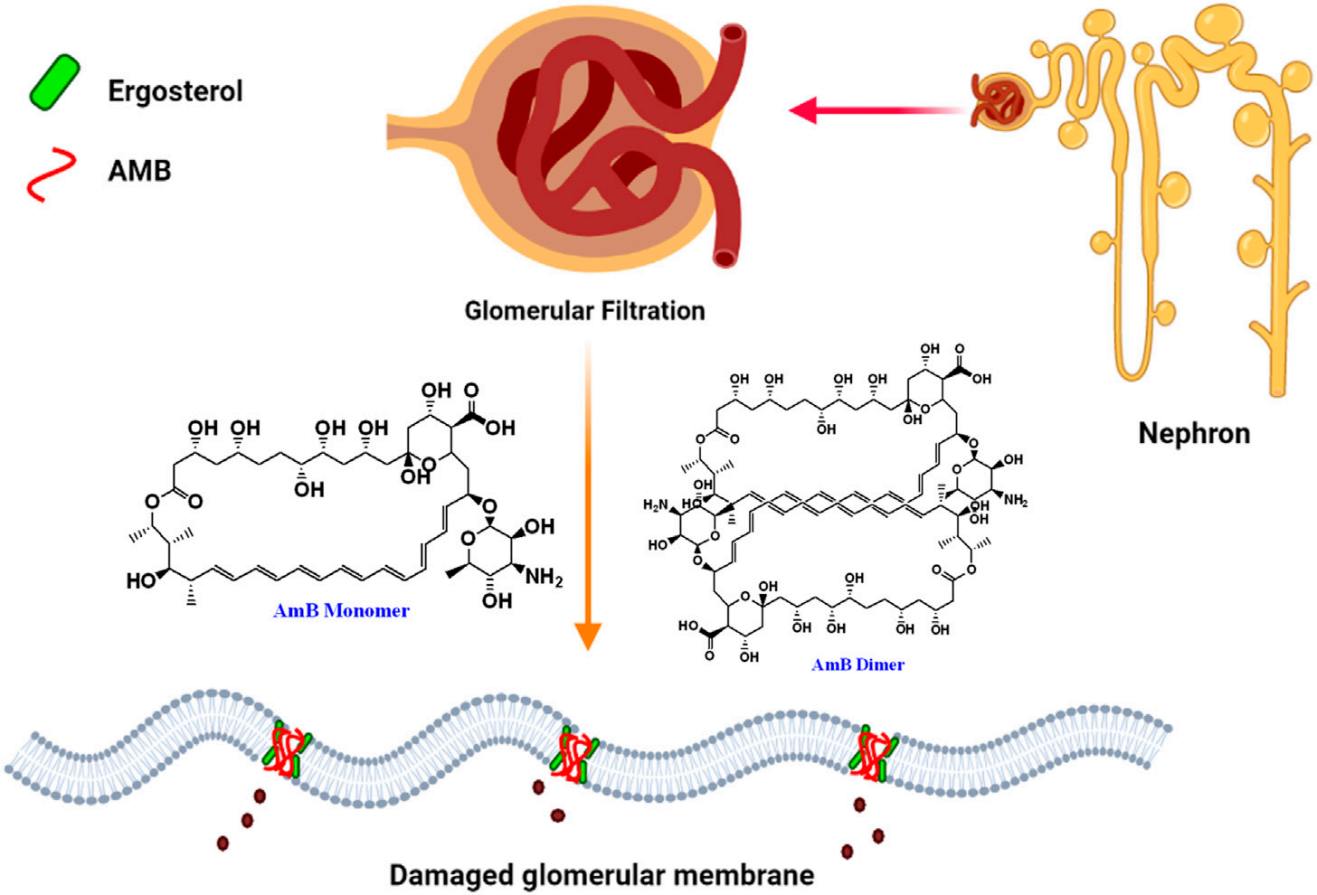
Pictorial presentation of AmB-mediated nephrotoxicity caused by ergosterol binding and depletion from glomerular membrane of nephron by dimeric insoluble form of AmB.

A study of 707 patients who received parenteral AmB deoxycholate therapy reported that 30% of the study population developed acute renal failure (Bates et al., 2001). Standard AmB deoxycholate associated nephrotoxicity is prevalent and is linked to a wide range of morbidity, the most serious of which is dialysis, resulting in a threefold increase in mortality, other medical complications and higher medical expenses. AmB dimerization/oligomerization was linked with renal toxicity. It is important to note that the liposomal formulation of AmB (AmBisome) which is composed of hydrogenated soy phosphatidylcholine, cholesterol, and distearoylphosphatidylglycerol along withother ingredients reduces the AmB toxicity but comes with high cost of treatment. As a result, novel approaches to reduceAmB-related nephrotoxicity are urgently needed (Gursoy et al., 2021). Most plausible mechanism of nephrotoxicity is demonstrated in Figure 2.

### Clinical status of liposomal AmB and its modifications in treatment of VL

From 2003 to 2005 three AmB-based lipid formulations with reduced toxicity than micellar formulations were used. The three formulations were Liposomal AmB (L-AmB) (Landi-Librandi et al., 2011), AmB lipid complex (ABLC) (Hachem et al., 2008), and AmB colloidal dispersion (ABCD), which contained AmB and sodium cholesteryl sulphate (Guo, 2001). AmB-deoxycholate lipid emulsion complex (ABLE) is a newly developed formulation and licenced in India (Sundar et al., 2014b).

This lipid formulation of AmB is the most effective and least harmful. One of AmBisome’s major flaws is its cost (Sundar et al., 2014b). However, due to the WHO pricing agreement, AmBisome is now only $18 per 50-mg vial in endemic areas (Sundar and Singh, 2018). In India, a single dose of AmBisome (10 mg/kg) or a short course of AmBisome plus miltefosine/paromomycin cured 95% of cases (Sundar and Chakravarty, 2010; Sundar and Singh, 2018). Bangladesh has also confirmed the efficacy of a single dosage of AmBisomeagainst VL (Ekram et al., 2021). However, poorer drug susceptibility against*L donovani* strains in East Africa necessitates doses greater than 20 mg/kg (Croft et al., 2006).

Although AmBisome is the preferred treatment for HIV/VL co-infection, it is more toxic and reinfections are more prevalent. Thus, efforts to discover effective secondary preventive treatments for HIV/VL patients continue. The need for a cold chain is also considered as a disadvantage when using AmBisome in rural places since long term storage in cold temperature is required for AmBisome’s efficcay (Lindoso et al., 2016).

Although low-cost AmBisome-like formulations exist, their effectiveness and safety profiles can differ dramatically from the original medicine due to manufacturing process changes. This problem demonstrates the enormous difficulties of large-scale nano-drug formulations manufacturing with robust reproducibility in efficacy. Therefore, regulations for showing bioequivalence for generic AmBisome medicines must be reviewed regularly (Croft et al., 2006; Desai, 2012; Lindoso et al., 2016).

Even though AmBisome is the preferred treatment for leishmaniases, relapses and clinical failures have increased in patients with HIV/VL co-infection, CL and MCL. Thus, further research is required to develop AmB based formulations that are more efficient, less hazardous, and less expensive. The finding of AmB resistance but not with AmBisome resistance in therapeutic isolates of *Leishmania donovani* in India allows this medication to continue but new developments of these liposomal formulations are in urgent need and constantly explored.

**Fungisome**-(L-AmBL) is a liposomal formulation of AmB in saline developed by an Indian company Lifecare Innovations (Gurgaon, Haryana, India). It is approved by the Drugs Controller General of India (DCGI) and has been available in the Indian market since 2003 (Sundar et al., 2015). The pricing of the new liposomal AmB formulation L-AmBL has to be competitive and cheaper than L-AmB The sealed bottles of L-AmBL were stored at 2–8°C and subjected to sonication before administration. Sonicated L-AmBL was stored for up to 24 h at 2–8°C and shaken well before use. Partially used vials were not stored for future patient use and were segregated for disposal. Every lipid formulation of AmB has different safety and efficacy, even with the same doses, and thus, it is necessary to test every new formulation as a new drug (Sundar et al., 2015).

L-AmBL is used in a 7-day regimen to treat VL to reduce lengthy hospitalization, expenditures, and non-compliance. In recent studies, VL patients received five or 7.5 mg/kg of L-AmBL or 10 mg/kg in two 5-mg/kg doses. All patients recovered after 1 month 6-Month cure rates for 5, 7.5, and 10 mg/kg total dosages were 60%, 50%, and 90%, respectively. After this success L- AmBL we tested a higher single dose. Poor countries can buy 50 mg vials of L-market AmB for US$18. All liposomal amphotericin B formulations, including L-AmBL, require a cold chain storage which makes them expensive (Goswami et al., 2020).

No L-AmBL-treated subjects reported nephrotoxicity in an Indian post-marketing trial. AmBisomenephrotoxicity affects 47% and 21% of neutropenic patients. 32% of AmBisome-treated invasive fungal patients had nephrotoxicity. 2% of VL patients administered with AmBisome10 mg/kg dose had nephrotoxicity, hepatotoxicity, anemia, and/or thrombocytopenia (Goswami et al., 2016; Goswami et al., 2020). One patient in the 10-mg group experienced temporary nephrotoxicity, while another in the 5-mg group developed reversible thrombocytopenia and acute pulmonary edema (Goswami et al., 2016). A bigger study is needed to explore the complete range of adverse effects. This clinical study implies L-AmBL is better and may replace AmBisome for VL. These L-AmBL clinical data reveal a superior treatment plan compared to single-dose AmBisome approach, which is still considered the best option for VL treatment. This Indian-made L-AmBL showed promise in phase three studies, but more research is needed to verify its usefulness and safety specially with a broader clinical trial involving people from different geographical regions (Goswami et al., 2016). Pharmacokinetic and dynamic comparison of AmB major formulation is listed in the Table 2.

**TABLE 2:** Pharmacokinetics and pharmacodynamics parameters are compared between most important formulation of AmB.

Property	L-AMB (AmBisome)	AmB-DOC (fungizone)
Molecular		
Chemical structure		
Structure composition	Uni-lamellar spherical liposomes	Micelles
Size (μm)	0.08	<0.04
Molecular weight (g/mol)	924	924
AmB:lipid ratio	1:9	Not required
Pharmacokinetic […….]		
CL (ml.kg/h)	9.7 ± 5.4	13.1 ± 2.0
Renal Clearance (ml h-1kg-1)	0.495 ± 0.25	4.1 ± 0.68
Vd (L/kg)	0.2–1.6	2–2.3
Cmax (mg/L)	22.9 ± 10 (2 mg/kg)	1.43 ± 0.2 (0.6 mg/kg)
Terminal half life -second phase (h)	6–23	10–24
AUC (0–24) mg.h/L	171 + 126	1–30
Distribution		
Organ distribution	Spleen > liver > kidney > lung	Liver > spleen > lung > kidney
Clinical status		
FDA approved Year		
Standard dosing in invasive leishmaniasis		
Nephro/Hepato-toxicity risk		
Cost		

## Most important physico-chemical properties of nanoparticles for drug delivery

### Shape/size of particles

It is possible to create NPs of various sizes and forms because different methods of synthesis/formulation allow this flexibility (Wilczewska et al., 2012). The toxicity and effectiveness of NPs are in turn determined by these variances in size or structures. Numerous studies have shown that the key factors of toxicity of NPs are associated with size, shape, charge, and surface coating. Therefore, it can be controlled to produce non-toxic NPs. For cellular uptake, interactions with the immune system, and, consequent, removal from the body is crucial for NPs (Borel and Sabliov, 2014). The parasite mostly affects the macrophages of liver and spleen in VL. As a result, investigations on the absorption of macrophage cell lines employing various NPs may offer a method for better drug delivery for VL (Basu and Lala, 2004). It was found that murine macrophages took up triangular NPs far more than the other two forms of NPs (stars, rods, and triangles) of the same size (Xie et al., 2017). These studies highlight the significance of NPs surface characteristics and geometry for transport over biological barriers.

### Surface charge and pH

Although cationic and anionic NPs are taken up by cells using similar methods, higher absorption rates are invariably linked to stronger biological effects. When incubated with fluorophore-conjugated polystyrene nanoparticles (F-PLNPs), phagocytic differentiated THP-1 cells or non-phagocytic A549 cells exhibit difference in uptake that is strongly linked with zeta potential of the NPs (Jeon et al., 2018). This finding indicates that surface charge is a crucial role in cellular absorption efficiency, even though other factors including aggregation, protein corona formation, which depends on surface charge, and compositional elements can also partially or indirectly affect cellular uptake. It has been observed that charged NPs opsonize more quickly than neutral NPs (Owens and Peppas, 2006). It has been observed that zwitterionic NPs have extended circulation after cellular entry. The surface charge of NPs affects cellular absorption, bioavailability, toxicity, and mucoadhesion or diffusion. The biocompatibility and absorption efficiency of NPs can change due to changes in surface functionality and charge (Sanità et al., 2020). Surface modifications utilising hydrophilic/hydrophobic components with variable charges enable drug release mechanisms following penetration through biological barrier.

After infection, Leishmania species reside in a low pH in acidic phagolysosomal compartment. The pH-dependent drug release will be crucial in the site-specific accumulation of pharmaceuticals since a drug moiety capable of reaching a target should resist the changing pH encountered across different tissues or subcellular compartments. Gold nanoparticles (GNPs) have been discovered to alter the pH towards basic range and lower lysosomal activity whereasorganic NPs have been reported to breakdown in a very acidic lysosomal environment (Manshian et al., 2018). Due to better stability and reduced toxicity at that pH, a chitosan and chondroitin sulfate-based NP delivery system with AmB is more successful than normal AmB at treating tegumentary leishmaniasis (Ribeiro et al., 2014). For treating CL, a biopolymer of polycaprolactone loaded with AmB has demonstrated superior efficacy to AmBisome at both a physiological pH of 7.4 and a lower skin-relevant pH of ∼5.5.

### Different nanoparticles based AmB drug delivery approach for treatment of VL

**FIGURE 3:**
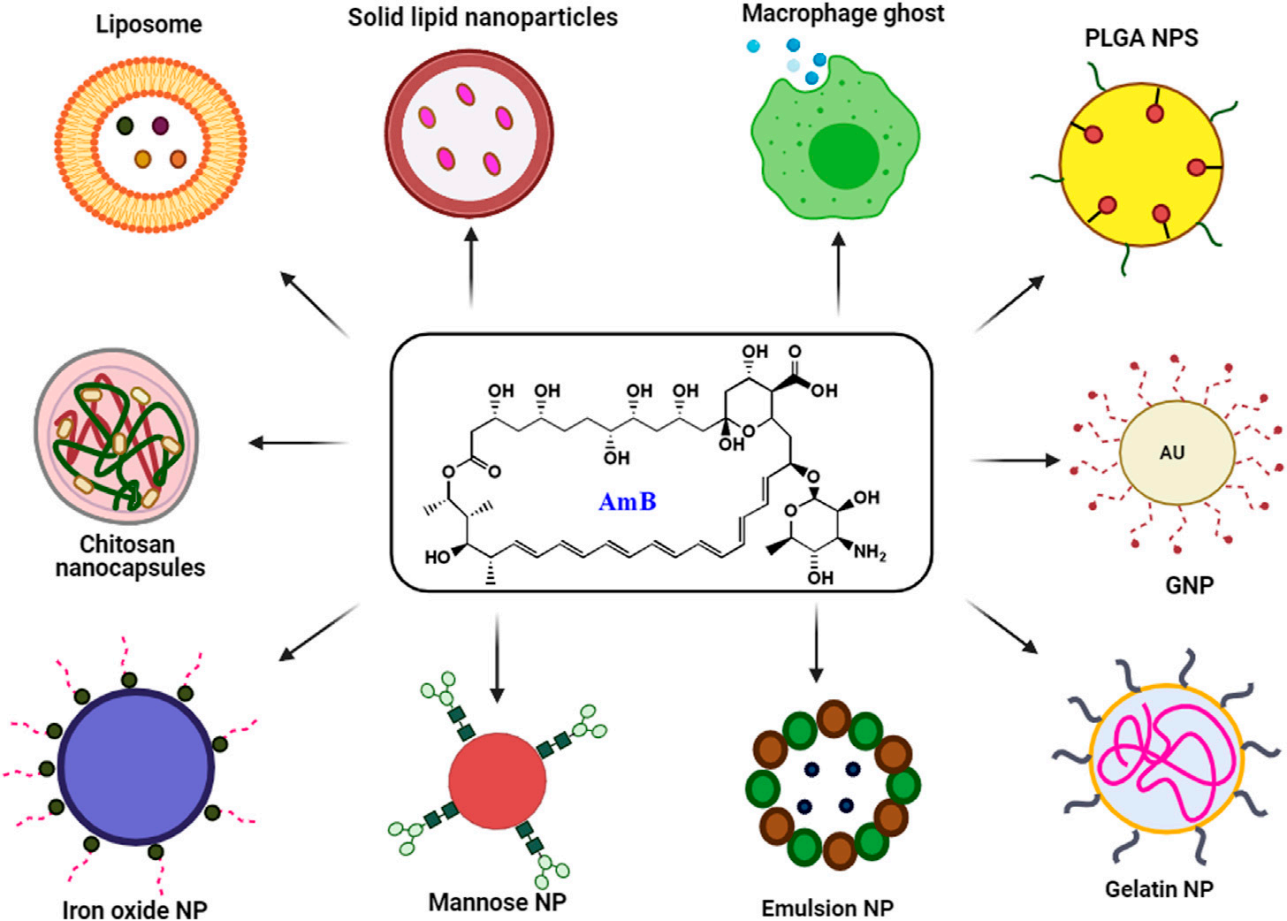
Schematic presentation of major nano formulation based delivery of AmB.

The use of existing therapies for treating leishmaniasis is limited due to toxicity, resistance, expenses, and administration issues with patience non-compliance. Recently, nanotechnology has emerged as a way to overcome current therapy limitations for diseases like leishmaniasis. Nanotechnology uses nanometer-sized materials to deliver, diagnose and cure infectious disorders (Jamshaid et al., 2021). Due to improvements in pharmacokinetic parameters such as absorption, distribution, metabolism, and excretion (ADME), bioavailability and bioequivalence (BABE) nano-drug delivery systems outperform traditional chemotherapy (Hamidi et al., 2013). Its high efficacy, low toxicity, higher bioavailability, sensitivity, specificity, sustained and controlled release on-target make NP-based delivery a better drug delivery systems (Hamidi et al., 2013). To improve the deliverymetallic NPs, polymeric NPs, solid lipid NPs (SLN), liposome-based NPs, nanocapsules and nanoemulsions were developed and used extensively (Patra et al., 2018). Pictorial representation of different AmB nano-formulation is demonstrated in Figure 3. All different NP based AmB drug delivery with its mechanism is listed in the Table 3.

**TABLE 3:** Nanoparticle based AmB delivery by different approaches for VL are listed with their most probable mechanism.

AmB nanoparticles	Materials	Target	Mechanism	References
AmBemulsomes (TLEs-trilaurin-based emulsomes)	Phosphatidylcholine, trilaurin and cholesterol coating with O-palmitoylmannan	LD infected macrophage	-	Gupta et al. (2007)
Amphotericin B-chitosan-coated Loaded Solid Lipid Nanoparticles	Stearic acid, palmitic acid and soya phosphatidyl choline (PC)	Macrophages/immunoadjuvant	ROS and TH-1 dependent inhibition of LD	Jain et al. (2014)
mannosylated oral amphotericin B nanoformulation	Chitosan, sodium tripolyposhphate (TPP), thioglycolic acid (TGA), d-mannose	Macrophage	increased macrophage uptake	Sarwar et al. (2018)
Alginate coated AmB lipid nanoconstructs	Hydrogeneted soya Phosphatidylcholine, cholesterol, stearylamine and sodium alginate	Macrophage	immunostimulant and chemotherapeutic activity	Singodia et al. (2011)
Peptide coated Iron oxide nanoparticles (GINPs) encapsulated amphoterecin B	Peptide (glycine), Iron oxide	Macrophage phagocytic	Improved efficacy by higher macrophage uptake of formulation	Kumar et al. (2017)
PhoS coated PLGA-AmpB NP	Poly (lactic-co-glycolic acid) (PLGA), 3-O-sn-Phosphatidyl-l-serine (PhoS), Tween 80, disodium phosphate, monopotassium phosphate	Selective Macrophage targeting	PhoS dependent identification	Singh et al. (2018)
VBS-AmB-solId lipid nanoparticles	vitamin B12-, stearic acid	Enhanced targetabilityto macrophage	Selective cellular death of LD	Singh et al. (2020)
AmB-encapsulated chitosan nanocapsules (CNC-AmB)	Chitosan, Soya lecithin, soya bean oil, Tween 80	-	Immunostimulant and chemotherapeutic activity, also prevent parasite resist phagocytosis	Chitosan, (2013)
surface functionalized gelatin nanoparticles (*f*-GNPs)	Gelatin Type A, glutaraldehyde grade I, trypsin, concanavalin A, *N*-hydoxysuccinimide (NHS)	Mannose receptors	Increased drug uptake-selective LD cell death	Nahar et al. (2010)
AmB-loaded poly (lactic-co-glycolic acid, PLGA) nanoparticles	poly (lacticco-glycolic acid), poloxamer 188, methanol: acetone mixture (1:2 v/v)	-	Based on loading and release	Verma et al. (2011)
modified dual drugloaded solid lipid nanoparticles (m-DDSLNs) (encapsulated with Amphotericin B and Paromomycin)	2-hydroxypropyl-β-cyclodextrin (HPCD),trehalose		enhanced the uptake of SLNs by the macrophages, reduction in liver parasite burden	Parvez et al. (2020)
Sodium Alginate Glycol Chitosan Stearate Nanoparticles	Glycol chitosan, sodium alginate, stearic acid, [1-ethyl-3 (dimethylamino) propyl] carbodiimide hydrochloride (EDC)		-	Gupta et al. (2015b)
Mɸ ghost nanoparticle	Macrophage	Macrophage membranesurface receptor	High drug uptake-caused the conc. Dependent cell death	Kumar et al. (2019b)
amphotericin B encapsulated PLGA-PEG nanoparticles	copolymer PLGA–b–PEG (poly (d, l-lactide-coglycolide)-block-poly (ethylene glycol))	Macrophage uptake	macrophage phagocytic	Kumar et al. (2015b)
AMB gold nanoparticle	HAuCl4, 0.05% trisodium citrate	Ergosterol	Increased ROS dependent cell death	Kumar et al. (2019a)
cationic stearylaminelipid-polymer hybrid nanoparticle	D-alpha-tocopheryl polyethylene glycol, stearylamine		Th-1 biased immune-alteration	Asthana et al. (2015b)
chitosan-chondroitin sulfate based nanodelivery	chitosan-chondroitin sulfate	CD163 receptor	Specific delivery to macrophage	
Calcium phosphate (CaP) nanoparticles	Calcium phosphate, Calcium chloride	Fcγreceptor	Induce a Th1-biased immune response	Chaurasia et al. (2016)
lipo-polymerosome (L-Psome) self-assembled Chitosan stearic acid copolymer	Glycol chitosan, stearic acid, cholesterol, α-phosphatidylcholine		upregulation of Th-1 cytokines anddownregulation of Th-2 cytokines	Gupta et al. (2014)
mannose-grafted amphotericin B lipid nanospheres	Phosphatidylethanolamine lipid nanospheres, egg lecithin, cholesterol	Mannose receptor	Reduced parasite burden in the liver and the spleen	Veerareddy et al. (2009)
liposomal amphotericin B KALSOME™10	liposomal lipid, phospatidylcholine, ergosterol	Macrophage	immunomodulatory activities	Asad et al. (2015)
HPMA–AmBcopolymer	*N*-(2-hydroxypropyl)methacrylamide (HPMA) copolymers, GlyPheLeuGly (GFLG) linker	-	-	Nicoletti et al. (2009)
Mannosylated chitosan nanocapsules	Mannose sugar-bound chitosan	Macrophage mannose receptors	improve selective delivery of AB into macrophages	Asthana et al. (2015a)
Lactoferrin-appended amphotericin B bearing nanoreservoir	poly (d,l-lactide-coglycolide), Lactoferrin	C-type lectin receptors	Chemotherapeutic andimmunomodulatary	Asthana et al. (2015c)

### Solid lipid nanoparticles

SLNs are solid lipid nanospheres stabilised by biologically suitable emulsifiers within a size range of 50 to 1,000 nm (Doroud et al., 2010). SLNs have the advantage of regulating the immune response when combined with chitosan (Doroud et al., 2010; Zolnik et al., 2010). Chitosan-coated SLNs are loaded with AmB (AmB-C-SLNs)to treat leishmaniasis. Macrophages absorbed AmB-C-SLNs are better than non-coated AmB SLNs. Also, the AmB-C-SLNs produced significantly increased TNF and IL-12 and decreased IL-4, IL-10, and TGF- β cytokines than standard AmB when tested against amastigote-infected macrophages. Solid lipid nanoparticles loaded with AmB and Paromomycin were shown to be the most efficient inhibitors against *Leishmania donovani* intracellular amastigote with >96% clearance of parasite burden (Parvez et al., 2020).

### Nano-emulsions

Cholesterol in nanoemulsions (CHOL-NE) increased the stability and performance of AmB encapsulation (Briuglia et al., 2015). The cytotoxicity of CHOL-NE-AmB was lower than that of AmB-deoxycholate (Santos et al., 2018). Thus, AmB-loaded cholesterol-stabilized NE was considered an improved antileishmanial formulation. Furthermore, when compared to AmBisome, AmB-incorporating microemulsions (ME-AmB) had slightly higher activity against intracellular LD amastigotes, but they were more cytotoxic to mammalian cells, resulting in a lower selectivity index (do Vale Morais et al., 2018). CopNEC-AmB, a nanoemulsified carrier device for oral delivery of AmB, was developed in search of synergistic effects between Copaiba oil (Cop) and AmB (Gupta et al., 2015a). The formulation was found to be stable in gastrointestinal fluids. Further, > 7- fold increased bioavailability was observed for Cop NEC-AmB than standard AmB. Histogical studies on rats indicate no kidney toxicity compared to fungizone.

### Mannose craft-based delivery

Prabhakar et al. developed mannose-grafted lipid nanospheres in 2009. This approach delivers drugs to macrophages *via* the mannose receptor. As an immunological stimulant for macrophage selective administration, AmB-loaded chitosan nanoparticles functionalized with mannose (LN-A-MAN) were utilised (Veerareddy et al., 2009). In the *Leishmania donovani* hamster model, mannosylated chitosan nanocapsules showed higher absorption *in vitro* and vivo. Tissue distribution shows LN-A-MAN goes rapidly in the liver and spleen and kills the parasite more efficiently (>95%) than fungizone (∼82%). LN-A-MAN-treated mice showed reduced toxicity as measured by decreased serum glutamate pyruvate transaminase (SGPT), alkaline phosphatase (ALP), urea, and creatinine levels as compared with Fungizone treatment.

### Lipid bilayer based AmB delivery

Liposomes are vesicle-like structures consisting of a phospholipid bilayer and cholesterol that are more effective at treating leishmaniasis (Veerareddy et al., 2009). The ability to improve drug stability, versatility to handle site-specific targets, sustained release of the drug from liposome and low cytotoxicity are all advantages of using this drug delivery system (Diro et al., 2015; Georgiadou et al., 2015). Liposomal amphotericin B as AmBisome is surprisingly the most efficient form of nanomedicine in use for leishmaniasis (Minodier et al., 2003). Paromomycin and pentamidine are another reference drug that was studied in their liposomal nanoformulations. (Banerjee et al., 1996; Picard et al., 2015).AmBisome, although very expensive, is currently the widely accepted nanoformulation for the treatment of leishmaniasis and also for fungal infections due to its reduced cytotoxicity compared to fungizone (Asthana et al., 2013a).

To create a lower-cost AmB liposome formulation than AmBisome^®^, a new formulation was developed in which two molecules of stigmasterol are covalently bound to glycerophosphocholine. The mice model data suggested similar pharmacokinetic profiling in serum and tissue as AmBisome does (Iman et al., 2011). Interestingly, inclusion of these two lipids increased the maximum tolerated dose of these formulation (DSHemsPC of particle size ∼100 nM) almost 6 times compared to AmBisome. AmB was encapsulated in the bilayer with 75% efficiency in the monomeric form. As a result, further research into this new liposomal formulation is required. This formulation is also evaluated on another leishmaniasis, where it was effective against *L. braziliensis* amastigotes similar to AmBisome^®^ (Iman et al., 2011).

### Macrophage targeted AmB delivery

Drug delivery combining specific NP-based ligand and macrophage receptor interactions are gaining prominence. This has led to many prospective cancer studies, but only one has been published in VL by Kumar et al., 2019. That study used a ghost cell method based on macrophage membrane-derived nanovesicles to transport and deliver AmB on the target. Macrophage ghost membrane proteins interact with the infected neutrophil-macrophage system and non-infected macrophages differently to disseminate infection in the host. In this ghost cell AmB carrier, these membrane proteins help to target delivery to infected organs. Compared to normal direct administration and antileishmanial therapy of AmBisome this low-cost, biocompatible delivery vehicle had reduced toxicity and lowered LD_50_ (Kumar et al., 2019b).

### Polymeric nanoparticle

Polymeric nanoparticles (PNPs) have gained substantial attention in recent years due to their unusual characteristics and behaviors (Mallakpour and Behranvand, 2016). The therapeutic agents can be encapsulated, uniformly dispersed (nanospheres) or enclosed by the polymeric membrane (nanocapsules), modifying the drug release profile in the human body. PNPs and liposomes are widely recognised as safe drug delivery vehicles in the pharmaceutical industry. It is widely employed due to its non-toxicity to biological systems, biocompatibility, storage stability, controlled release, and on-target delivery (ud Din et al., 2017). PNPs are solid colloidal nanodevices (Bolhassani et al., 2014). It is important to choose a polymeric material that is compatible with the compound to be provided, has the desired morphology of the nanosystems (capsules or matrixes) and can modify the surface of nanoparticles through chemical methods or physical contact with specific molecules (ud Din et al., 2017). PNPsare also considered as a viable approach for oral administration of water-insoluble molecules (Mallakpour and Behranvand, 2016). These NPs outperform liposomes in terms of stability in harsh condition of preparation, storage, and by having longer stability in biological fluids (ud Din et al., 2017). This approach boosted aqueous solubility of the drug thereby increasing macrophage internalisation. PNPs were explored as nanocarriers for antileishmanial substances because they can be internalised into infected cells by enhancing the entrapped substance’s pharmacological impact (Palma et al., 2018).

Polymers including polylactic acid (PLA), polyglycolic acid (PGA), polylactide-co-glycolide (PLGA), polycaprolactone (PCL), and polycyanoacrylate (PCA) are utilised to build polymeric drug delivery systems (Liu et al., 2008). Encapsulated AmB with TPGS (P-GP efflux inhibitor) exhibits better oral bioavailability and less nephrotoxicity (Italia et al., 2009; Tu et al., 2012). Antileishmanial chitosan and alginate polymers have also been explored (Ribeiro et al., 2014). They can produce more efficacious antileishmanial agents (Asthana et al., 2013b).

Macrophage (J774.1) internalized 20-fold more AmB/M-Cs-coated Poloxamer 407 micelles. Compared to uncoated micelles (Singh et al., 2017). When treated with AmB/M-Cs *via* intra-peritonial (IP), LDinfection was dramatically reduced. *In vitro* and *in vivo*, chitosan coated AmB generated a better Th1 defensive immune response, implicating immuno- and chemotherapeutic actions. Bose et al., 2016 showed that chitosan and chondroitin sulphate-based AmB medicines can be surface-modified to target LD-infected macrophages.

### Metallic nanoparticles

Metallic NPs are playing very promising role in the field of nanotechnology because it has wide variety of application in developing of nanomedicine including delivery, biosensors, imaging *etc.* (Patra et al., 2018; Vahedifard and Chakravarthy, 2021). The properties of these NPs like size, shape and high surface area to volumes makes it suitable for many biological application including antileishmanial therapy and for treatment of several other diseases (Salata, 2004; Chowdhury et al., 2017). Currently, biological method is found most suitable for synthesis of metallicNPs and more research is focusing on that because several disadvantages associated with traditional methods like chemical and physical methods of synthesis (Vahedifard and Chakravarthy, 2021). The properties of GNPs including biocompatibility, low toxicity and suitability of preparation in any size/shape makes them suitable for various application (Yang et al., 2021). Prakash et al. synthesized GNP conjugated with Amphotericin B (GL-AmB) and showed that IC50 of GL-AmB was reduced ⁓5 fold and ⁓2.5 fold against amastigote and promastigote respectively as compared to Amphotericin B (Kumar et al., 2019a). Also flavonoid functionalized gold nanoparticles was shown very effective against wild and resistant type strain with low toxicity and high selectivity index (Prasanna et al., 2021). The flavonoid 7,8-dihydroxyflavone (DHF) in association with GNP showed significant inhibition of arginase activity of polyamine biosynthesis pathway to kill the parasite. Adil et al. (Allahverdiyev et al., 2011) showed silver nanoparticles (AgNPs) having enhanced antilesihmanial activity against L*. tropica* by inhibiting proliferation and metabolic activity of promastigote by 1.5-3 fold respectively in dark condition and 2–6.5 fold respectively under UV light irradiated condition. Also it has significant effect on amastigote killing during host-pathogen interaction under UV light. Isatistinctoria mediated amphotericin B bound AgNP also showed strong antileishmanial activity with 96% inhibitory potency under visible light and found more efficient strategy for treatment of CL (Ahmad et al., 2016).

### Target specific delivery

Leishmaniasis is an intracellular infection in which the parasite is housed in the phagolysosomal compartment of a macrophage in the deeper tissues. Using a guiding molecule on the surface of NPs, specific drug delivery to macrophage infected cells could be achieved. Drug vehicles containing drug particles should be efficient enough to enter parasitized macrophages and de-load the drug at a concentration sufficient for antiparasitic effect. When tagged to a liposomal surface with Tuftsin (Thr–Lys–Pro–Arg), a naturally occurring macrophage activator tetrapeptide, the liposomal- Tuftstin results in increased anti-leishmanial activity. Since mannose ligands can bind to the C-type lectin receptors (CLRs) found on the surface of many pathogens, they have also been used for selective targeting (Rathore et al., 2011; Vázquez-Mendoza et al., 2013) Likewise, mannose-bearing PLGA nano/microparticles and mannose-coated lipid nanoparticles of AmB have shown higher liver uptake (Veerareddy et al., 2009). Other agents used to adjust drug delivery vehicles for selective targeting of macrophages includes chitosan, lactoferrin (Lcf) (Asthana et al., 2015a), anti-CD14 antibodies (Kumar et al., 2015a), and sodium alginate. Macrophages have receptors that allow them to uptake theseligand containing nanoformulation in leishmaniasis-infected macrophages selectively, and they outperformed normal AmB/drug-based delivery against both promastigotes and amastigotes.

Hemoglobin -guided nanocarrier was discovered to improve drug targeting specificity in infected macrophages. As a result, the IC_50_ for promastigotes and LD_50_ for intracellular amastigotes were lower with this biodegradable, low-cost AmB-loaded Chitosan–Chondriotin Sulphate (Cs-Chs) nanocarrier compared to traditional AmB therapy. When compared to a comparable dose of pure AmB, the toxicity profile of Cs-Chs-Hb-AmB was found to be favorable.

### Other formulations

Nanosized lipid nanoparticles of AmB stabilized with soya phosphatidylcholine (PC) and functionalized by macrophage-specific ligand (O-palmitoyl mannan, OPM) was formulated in trilaurin-based emulsomes for the treatment of visceral leishmaniasis (Gupta et al., 2007). Similarly, 3-O-sn-Phosphatidyl-l-serine (PhoS) linked PLGA nanoparticles were developed for specific targeting of macrophages. Phos triggers the high uptake by macrophages with increased drug concentration inside the targeted macrophages that led to high efficacy as compared to the traditional method. *In vitro* data showed ∼82% parasite inhibition as well in high *in-vivo* antileishmanial activity. Due to increased biocompatibility it has a high safety profile compared to marketed formulations (Singh et al., 2018). Further, mannose-anchored thiolated chitosan (MTC) based nanocarriers for enhanced permeability, improved oral bioavailability, and anti-parasitic potential of AmB. Toxicity studies suggest that this formulation had significantly reduced toxicity in comparison to only AmB. Further, it can be administered orally (Sarwar et al., 2018). In another study, peptide (glycine) coated iron oxide (Fe_3_O_4_) nanoparticles (GINPs) encapsulated AmB was used against VL. The results revealed that AmB loaded GINPs is ∼2 fold more effective than AmB and therefore, it has the promising role for use against VL (Kumar et al., 2017). For oral delivery of AmB, vitamin B_12_-stearic acid (VBS) based SLNs are synthesized with AmB encapsulation. It is prepared by using a combination of double emulsion solvent evaporation and thermal-sensitive hydrogel techniques. *Ex-vivo* studies in amastigotes showed improved efficacy (∼94%) with negligible toxicity in the J774A.1 cell (Singh et al., 2020). To enhance the stability and immunomodulatory activity of AmB, a new formulation was introduced with high ergosterol content with liposome KALSOME™10. KALSOME™10 increased the levels of IFN-γ and decreased the IL-10 secretion from both CD4 (+) and CD8 (+) subsets of T cells, as well as from culture supernatants of splenocytes, compared to that of normal, AmB and AmBisome treated animals (Asad et al., 2015).

### AmB role in fungal diseases

AmB is developed as an antifungal initially but that is later used to treat leishmaniasis, cryptococcal meningitis in HIV-infected individuals, and fungal infections in neutropenic patients. AmB exhibits strong *in vitro* action against a wide variety of fungal species. For more than 50 years, it has been used to treat invasive fungal infections. It is highly effective against the many of the Candida species, including *Candida albicans*, *Candida krusei*, *Candida tropicalis*, and *Candida parapsilosis*. AmB lipid complex (ABLC, Abelcet^®^) is highly effective against *candida* species related disease, further Liposomal AmB(L-AmB, AmBisome^®^) successfully used as an alternative and safe option of treatment. Apart from this AmB colloidal dispersion (ABCD, Amphotec^®^/Amphocil^®^) is well tolerated and effective treatment option for fungal infections.

### Future prospective on other nano-based delivery

Quantum dots (QDs) are nanoscale semiconductor crystals that have the ability to glow or fluorescence brightly when excited by a light source such as a laser. They usually range in size from 1 to 10 nm and smaller than standard NP-based delivery systems. Quantum dots are a new form of the fluorescent probe that can be used for biomolecular imaging, cellular imaging, drug delivery, and drug mechanism visualisation. Because of the simplicity of this technology, which has been widely used for anticancer studies, it can be used as a drug delivery vehicle, either naturally or as a QD conjugated drug, like AmB, to see its potential over conventional liposomal and NP-based delivery of AmB (Chand et al., 2021).

Till date there is no oral treatment for VL other than MTF which shows teratogenicity. Due to success of AmB, it is continuously explored in an oral delivery route. Increasing AmB dissolution in the digestive tract, improving lymphatic absorption, reducing drug degradation in the stomach’s acidic pH, increasing gastrointestinal transit, and finally targeting the formulation in organs of interest and minimizing off-target side effects are all necessary when designing oral AmB drug delivery systems. AmB is difficult to deliver orally due to its low solubility, permeability, and instability in gastric acidic pH (Parvez et al., 2021). In order to achieve effective plasma and tissue concentration after oral administration a variety of delivery strategies, including polymeric and SLN, micellar dispersions, cochleates, nanosuspensions, and lipid-based nanomedicines, have been reported with varying degrees of success (Thanki et al., 2019; Parvez et al., 2021; Thanki et al., 2021; Ramos et al., 2022). In some cases, similar tissue accumulation is seen following oral administration. This is why it is crucial to measure the levels of AmB in target organs like the liver and spleen for VL and the lungs, kidneys, brain, and liver for fungal infections when evaluating an AmB oral formulation. By improving the oral based drug delivery for AmB may provide an better delivery system and treatment for VL in near future.

## Conclusion

The advantages of nanotechnology are commonly recognized in the treatment of a variety of diseases including cancer, viral, bacterial and fungal diseases but rarely for parasitic diseases. Since, AmB is the key molecule for VL treatment it is likely nanoformulation of AmBhave a good future for VL treatment. There is a limitation in the current therapeutic of AmBisome^®^ due to its high cost although it has reduced toxicity and increased efficacy than other antileishmanial drugs. The NP-based delivery has advantages, as they were able to improve pharmacokinetic parameters including systemic bioavailability by acting as a drug carrier and deliver the payload on-target. Ability to produce different NPs with variability in size, charge and surface functionality allows drug with different physiochemical properties to be associated with NPs efficiently. This allows the drugs to be delivered with high efficacy at lower dose and, also, using specific targeted delivery combining receptor–ligand interactions. Nanocarriers were discovered to enhance AmB distribution orally or topically, and drug nanocarriers with targeted receptor and immunomodulatory components on macrophages are continuously explored. Based on these advances, therapeutic developments for leishmaniasis delivery using a QD-based approach and low-cost lipid-coated AmB delivery for oral delivery, as a better alternative for AmBisome, is anticipated shortly.
